# On Pain of the Back in Remittent Fever

**Published:** 1841-02

**Authors:** 


*On Pain of the back in remittent Fever.*—Dr. Kreamer, of Aix-la-Chapelle, in a work on intermittent Fever pnblished in 1837 asserts, that in all the intermittent fevers he has had an opportunity of observing, there exists at the situation of the first dorsal vertebra, a more or less severe pain on pressure. In order to perceive the symptoms, the pressure must be made from behind forwards, with the fingers upon the spinous process of the individual vertebra, not upon several vertebræ together; for in this latter case one bone is less affected in its relations with the others than in the former. If the intermittent fever is considerable, or old, or masked, pressure on the first dorsal vertebra, by giving pain, will suffice to evince the existence of fever. In simple or mild cases, the pain may be but trifling, and may require, particularly in phlegmatic individuals, that the attention may be directed to it; and in order to render it evident, pressure should be first made on the first cervical vetebra, then on the last dorsal, und then lastly on the first dorsal, when the patient may be asked if he feels any difference in the sensations produced. The pain exists during the paroxysms, as well as in the apyretic interval, is stronger in epidemic than in sporadic intermittent fever, exists in all forms, and continues during the sequelæ,

Dr. Grossheim has examined fifty cases of intermittent, with a view of testing the correctness of the above opinion, and the following are the results of his observations:—"1st, pain on pressure on some part of the spinal column is a constant symptom of intermittent fever, except in those cases in which the ligaments of the vertebræ have become by age or disease so rigid that they will not yield to pressure. 2. There is no definite locality to this pain; it may be situated in any part of the column, but is most frequent in the middle of the dorsal portion, especially in quotidian intermittents. 3. The extent of the pain also varies considerably ; one or two vertebræ only may be tender ; and the pain rarely occupies the space of more than five or six ; it may also be situated at distant parts, with intervals in which none is excited by pressure. 4. The intensity of the pain is equally variable. Sometimes it was so severe that the slightest touch of one of the spinous processes produced severe suffering ; but sometimes violent pressure was required to detect it. Among those vertebræ that excited pain when pressed, the middle one was commonly the most sensitive ; and in those above and below it, the tenderness gradually decreased. 5. The pain was more severe during the paroxysms than in the intermissions. When the severity of the fever diminished or the tendency to its returns grew less, the severity and the extent of the pain in the back also decreased; but the complete removal of the fever was not always accompanied by the entire loss of the pain, which often continued in a modified degree after the fever had ceased to return, and remained the longer the more severe it had previously been. 6. Complications of the intermittent fever did not appear to have any influence on the pain ; it continued when the character of the fever was altered either for the better or for the worse, and it returned in cases of relapse.

From observing the constant resistance of this symptom, the author was induced to try what would be the effect of remedies that would tend to correct the local excitement that seemed to exist. He relates very briefly five cases, in which eight or ten leeches were applied over the spine in the situation where pressure gave the most pain. In four of these no other remedy was required ; the pain ceased in a few days, and there was no recurrence of the febrile paroxysm.—*British and Foreign Med. Rev., July* 1839, *and Oct.* 1840. *Am. Jour. Med. Sci. for Jan.* 1841.

